# Myelomeningocele Closure: An Embryological Perspective

**DOI:** 10.7759/cureus.9682

**Published:** 2020-08-12

**Authors:** Mark Dias, Jessica Lane

**Affiliations:** 1 Department of Neurosurgery, Penn State Milton S Hershey Medical Center, Hershey, USA

**Keywords:** myelomeningocele, spina bifida, craniospinal dysraphism, embryology, neural tube defect

## Abstract

Myelomeningoceles (MMCs) represent a localized failure of primary neurulation during the fourth week of embryonic development. There are a number of misconceptions concerning the proper identification, classification, and surgical repair of these lesions. To provide surgeons with a working knowledge of early neural embryology as it relates to MMC closure as a localized failure of primary neurulation. We review the embryology of early neural development as a means of providing neurosurgeons with a better understanding of MMC closure techniques. Early neural development predicts the anatomy of MMC and knowledge of embryology helps guide repair. Repair of MMC is enhanced by knowing early neural development.

## Introduction

Although the repair of a myelomeningocele (MMC) is generally considered a ‘straightforward’ operative procedure, neurosurgeons sometimes misunderstand basic principles regarding the identification and operative repair because they fail to understand the underlying embryogenesis of these malformations. Other publications, while providing technical operative nuances, do not fully address the importance of embryological principles in MMC closure [1,2]. We therefore felt that an embryologically based operative review would help clarify these misconceptions and provide neurosurgeons with a reference with which to guide repair.

## Technical report

Normal embryogenesis and the embryology of MMC

MMC represents a failure of primary neurulation; among embryologists it is referred to as a neural tube defect (NTD). A practical review of the embryology of MMC is therefore vital to understanding its morphology and surgical repair. There are more complete references for those wishing a more comprehensive review of early neuroembryology [3,4].

**Figure 1:**
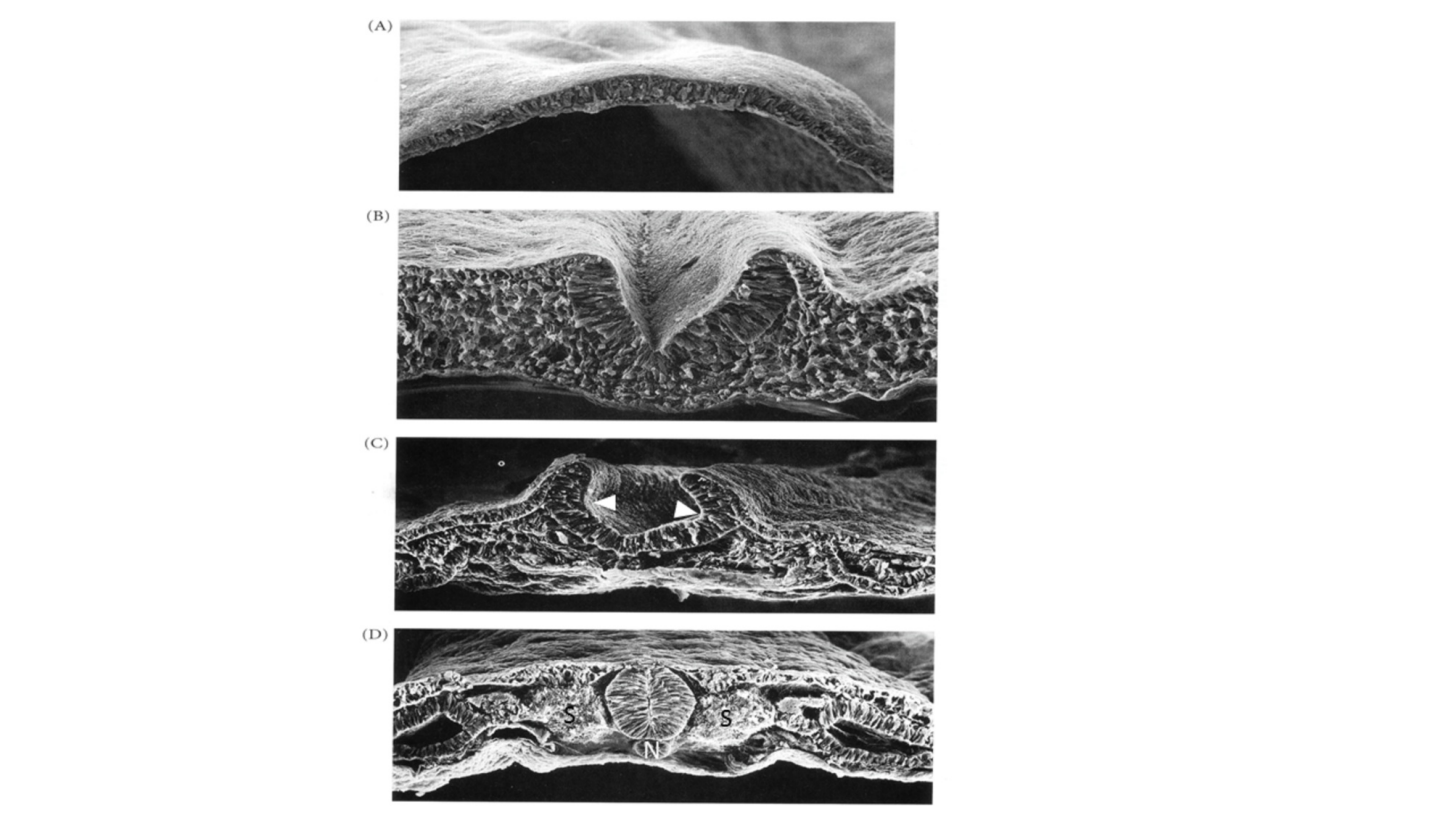
Scanning electron micrographs of chick neuroepithelium undergoing primary neurulation A. Initially the neuroepithelium is a flat sheet of pseudostratified cells attached around the periphery to the prospective cutaneous ectoderm (future skin). B. The midline neuroepithelial cells immediately overlying the notochord undergo cell shape changes that result in elevation of the neural folds on either side and the creation of the midline neural groove. C. Slightly later, similar cell shape changes occur in the dorsolatereal hingepoints (white arrowheads) that result in convergence of the neural folds toward the midline. Note that the edges of the neural folds remain attached to the adjacent cutaneous ectoderm at this stage. D. Completed primary neurulation – the neural folds have fused across the midline to create the closed neural tube overlying the notochord (N); the cutaneous ectoderm has separated from the neural folds (dysjunction) and has fused to form the midline skin overlying the neural tube. Cells from the adjacent somites (S) will migrate medially to surround the neural tube and will form vertebrae ventrally and posterior spinal arches posteriorly. Reproduced with permission from Gilbert, SF: Developmental Biology (7th Ed), Sinauer Associates, Sunderland, MA (page 394).

By the fourth embryonic week, the ectodermally derived neuroepithelium is a flat sheet of stratified columnar epithelium, connected around its edges to cutaneous ectoderm (which will form skin) (Figure 1A). During primary neurulation, morphological changes in midline neuroepithelial cells produce a midline furrow, the neural groove that overlies, and is induced by, the notochord (Figure 1B). These morphological changes include contraction of apical microfilaments and translocation of the nucleus to the basal region of the neuroepithelial cells overlying the notochord, resulting in a conformational change from columnar to trapezoidal shapes. Together with simultaneous changes in the adjacent cutaneous epithelium, these cell shape changes elevate the paired neural folds (Figure 1C). Slightly later, similar morphological changes produce paired dorsolateral hingepoints that lead to the convergence of the neural folds toward the midline (Figure 1C). To use an analogy, neural fold elevation is akin to raising ones arms at the shoulders, and subsequent convergence to bending the elbows to bring the hands together above the head.

The final event in primary neurulation involves fusion of the apposed neural folds across the midline to form a closed neural tube (Figure 1D). Simultaneously the adjacent cutaneous epithelium, which heretofore had been contiguous with the neuroepithelium, separates from the neuroepithelium (called dysjunction) and fuses with its contralateral member to form the dorsal skin. Contemporaneously neural crest, derived from neuroepithelial cells at the junction of neural and cutaneous epithelia, migrate away from the neural tube to form the dorsal root and dorsal root ganglia (as well as many other structures). Later adjacent mesodermal cells will migrate medially to surround the now-closed neural tube and will form meninges, veretebrae, and paraspinous muscles; notochordal remnants remain as the nucleus pulposus of the intervertebral disc (Figure 1D).

**Figure 2:**
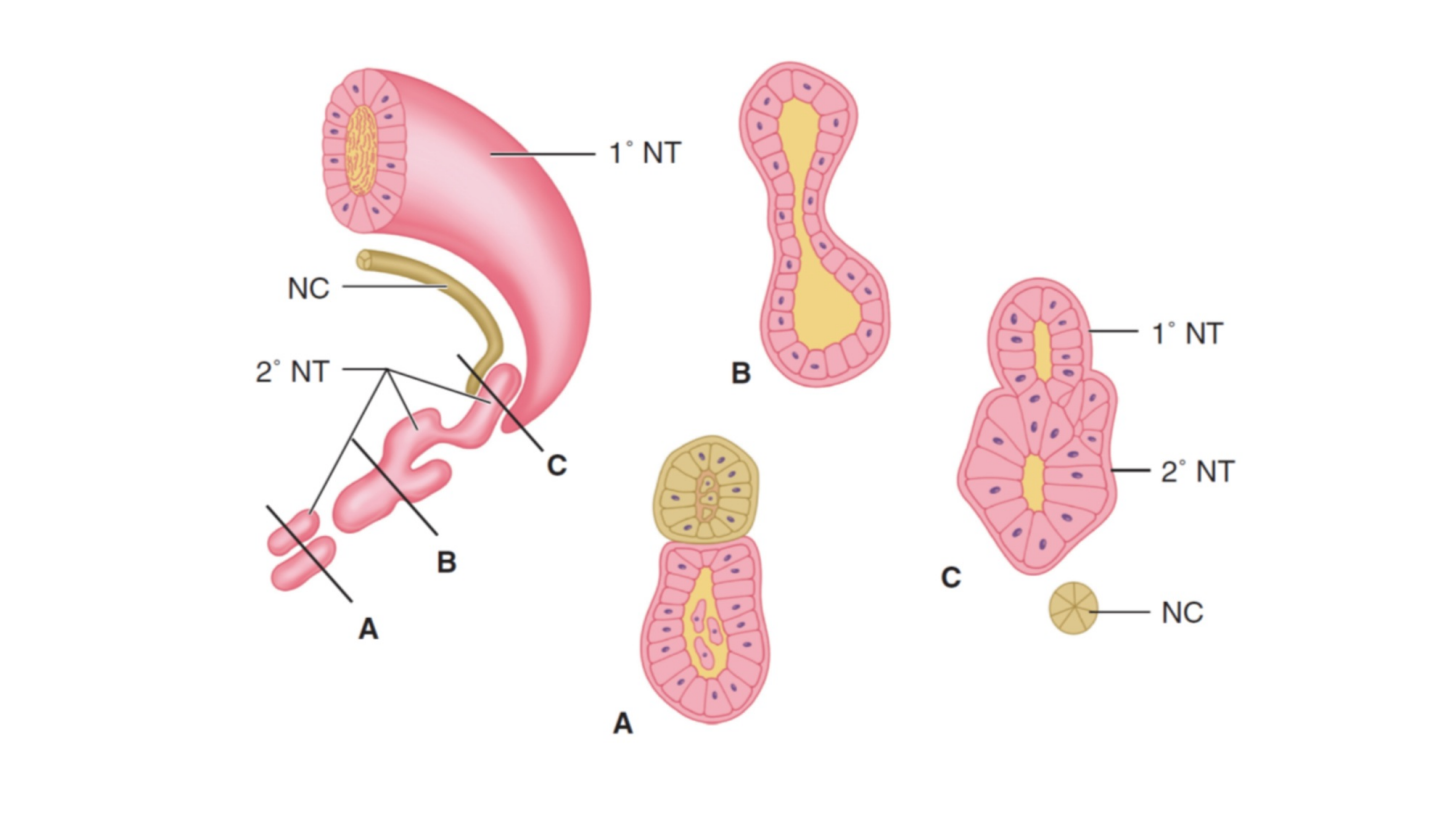
Secondary neurulation A. In the chick, cells from the caudal cell mass form tubules; these will eventually fuse to form a single large secondary neural tube (2o  NT), which will then fuse with the neural tube formed from primary neurulation (1o NT). B. In the mouse, cells are progressively added on to the caudal end of the 1o NT from the caudal cell mass (CCM) to form the 2o  NT. NC: Notochord. Reproduced with permission from Dias MS, Rizk EB: Embryology of occult spinal dysraphism. In Tubbs RS, Oskouian RJ, Blount J, Oakes WJ (eds) Occult Spinal Dysraphism. Springer 2019.

Primary neurulation creates the neural tube from the forebrain to the second sacral spinal cord segment (S2). The final portions of the neural tube to close are called neuropores; the posterior neuropore is located at S2. As primary neurulation is finishing at the end of the fourth week, a second process, secondary neurulation, is beginning. Secondary neurulation forms beneath an intact cutaneous epithelium. Cells of the caudal cell mass coalesce to form multiple tubules; these tubules subsequently fuse one with another to eventually create one large tubule, which then fuses with the neural tube derived from primary neurulation to generate a single neural tube (Figure 2). Secondary neurulation forms the caudalmost portion of the conus medullaris below S2 and the filum terminale. It is important to understand that secondary neurulation can occur independent of primary neurulation and therefore it is possible for a filum terminale to be present in patients with MMC.

**Figure 3:**
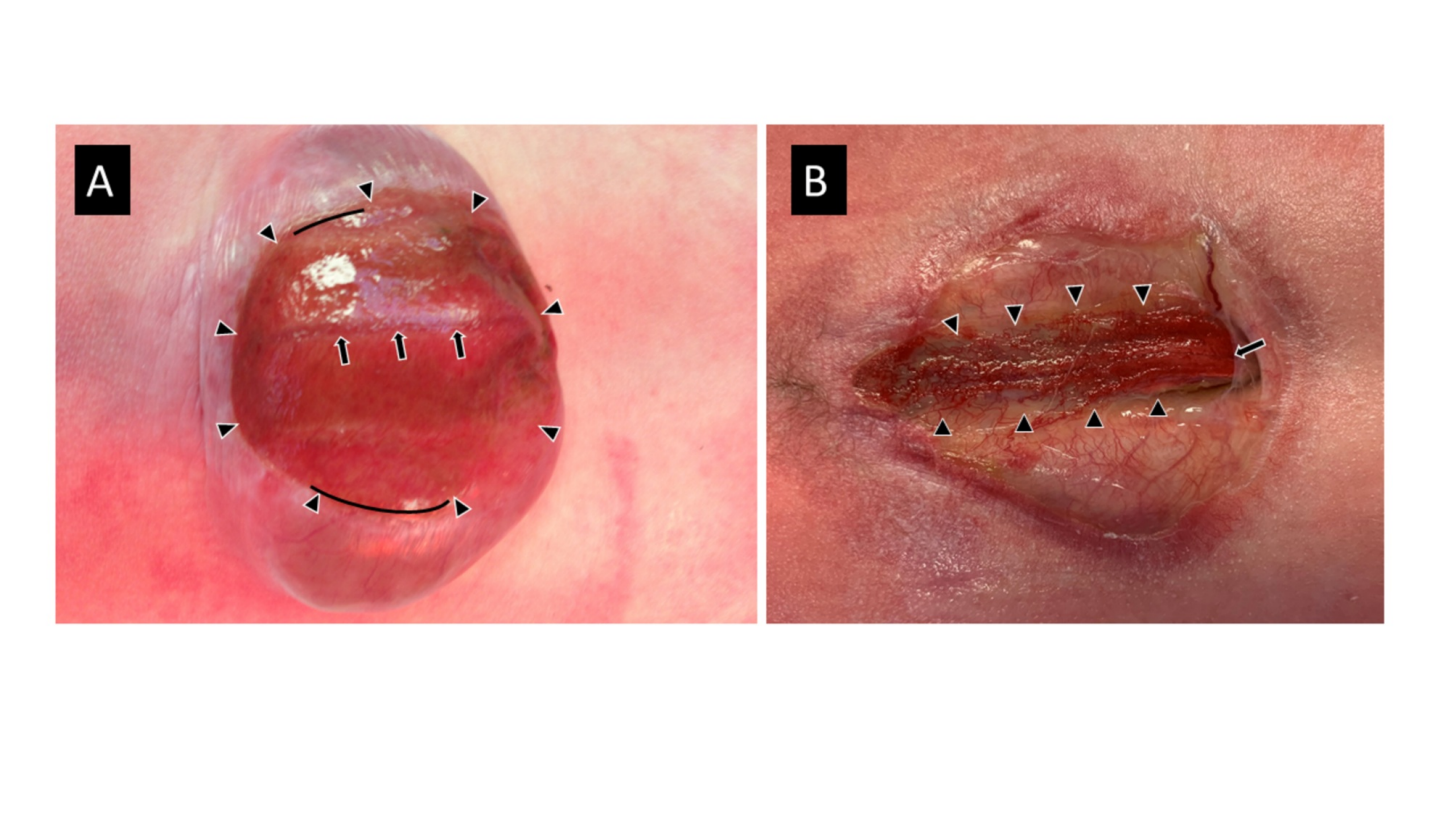
Types of myelomengingoceles A. Traditional myelomeningocele with neural placode (arrowheads) lying on top of a fluid filled sac (‘cele’). B. Traditional myeloschisis, with neural placode (arrowheads) lying flat within the spinal canal with no underlying fluid filled sac. In both instances, the neural placode is surrounded by, and attached circumferentially to, the surrounding dysplastic skin. Although not seen in A, the spinal cord in B can be seen exiting from the under the arachnoid (arrow) to become the neural placode.

Myelomeningoceles form through a localized failure of primary neurulation, usually through a failure of neural fold convergence; the midline neural groove is often still visible within the neural placode (Figure 3). Because primary neurulation has failed, myelomeningoceles are, by definition, open malformations. The neural placode is attached to the adjacent cutaneous epithelium around its borders, and the central spinal canal is open dorsally at the cranial end of the placode. However, spinal fluid leakage from the central canal is variable (perhaps because the central canal is occluded), leading clinicians to mistakenly identify some as ‘closed’ malformations when, in fact they are not.

Since the neural tube has not properly closed, the adjacent tissues that would otherwise subsequently surround the neural tube to form arachnoid, dura, and posterior vertebral arches are prohibited from doing so. Instead, they remain ventrolateral to the placode, and everted (much like a lobster tail). One result is that the subsequent formation of cerebrospinal fluid (CSF) is restricted to the space beneath the placode, elevating the placode on a ‘bubble’ of CSF (the ‘cele’ of myelomeningocele) (Figure 3A). If there is a mechanical tear in the thin cutaneous tissues surrounding the placode, CSF can leak through the tear, the ‘cele’ will collapse, and the placode will lie flat on the back (Figure 3B). The term myeloschisis has been used to refer to such a ‘flat’ placode, implying that it is somehow an embryologically different malformation. However, a myeloschisis is embryologically identical to a myelomengingocele, the only difference being mechanical changes that occur after the formation of the placode. McLone has therefore recommended that the term myeloschisis be eliminated, and all such malformations be referred to as myelomeningocele to avoid confusion [4].

Myelomeningocele repair

Understanding the embryology of the NTD is the key to understanding operative myelomeningocele repair. Whether flat or elevated, the first operative charge is to identify the neural placode. This is usually obvious, but there are times where the attachment of the placode is so minimal that it is difficult to visualize (and contributes to the misidentification of the lesion as a ‘closed’ myelomeningocele). Once the placode is identified, closure begins by making an incision immediately lateral to the placode into the thin and dysplastic epithelium (Figure 3A). Care should be taken to making the incision as close as possible to the placode to avoid leaving epithelial skin cells in the closure that can form a dermoid cyst [5]. However, because the placode contains both dorsal and ventral nerve roots that traverse subjacent to the surrounding skin, care should be taken to avoid cutting these roots during the initial dissection. The separation of the placode from the adjacent tissues proceeds circumferentially; at the superior end of the placode, the edges of the neural placode will converge toward one another as they approach the more rostral closed neural tube. Once the placode has been completely separated from the adjacent tissues, the edges are brought together and re-approximated by sewing the pial edges on each side to recreate the neural tube (Figure 2B). Although it may not reduce the incidence of subsequent retethering, this step of the closure makes subsequent untethering easier to perform.

**Figure 4:**
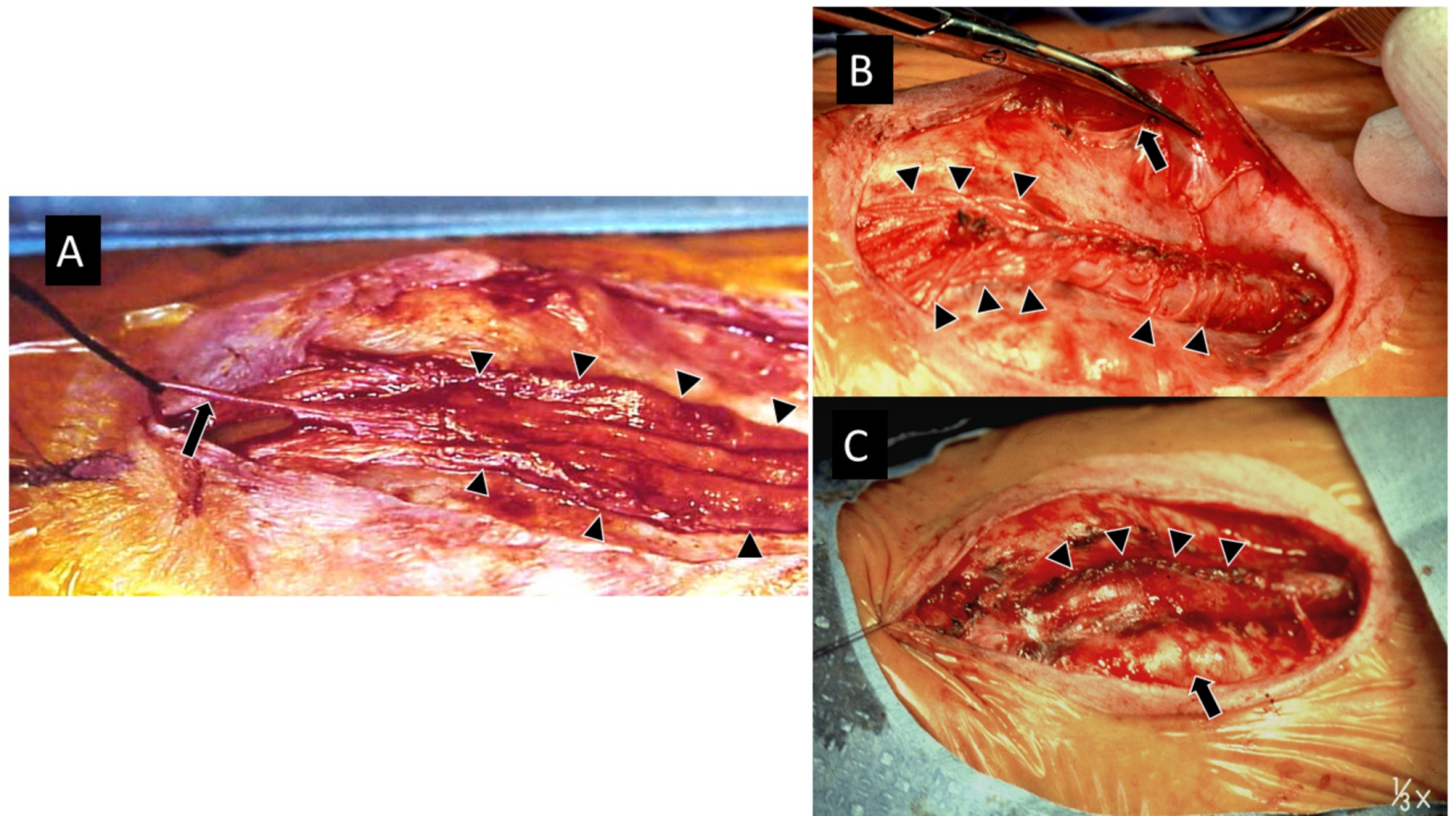
Closure of MMC A. The neural placode (arrowheads) has been dissected circumferentially from the surrounding skin. An intact filum terminale (arrow) is attached to end of the placode. B. The neural placode has been reapproximated in a pia-to-pia fashion to recreate a tubular structure. Note: abundance of nerve roots projecting from the placode (arrowheads). An incision has been made in the surrounding arachnoid/dura (arrow); the scissors have been inserted into this initial arachnoid/dura incision with one limb above and one below the tissue, and drawn laterally toward the edge of the arachnoid/dura before cutting it. This ensures a large enough dural sac. C. The closed dural sac (arrowheads). The surrounding paraspinous musculature (arrow) can optionally be elevated and brought medially over the dura. The skin is then undermined above the lumbodorsal fascia and closed. MMC = myelomeningocele.

Since secondary neurulation can proceed independent of primary neurulation, a filum terminale is occasionally present caudally and, if present, should be sectioned to eliminate another source of tethering (Figure 4A). Additionally, a split cord malformation (SCM) may be present cranial to, at, or caudal to the placode and, if present, should also be addressed [6,7]. Rarely, a hemimyelomeningocele may be present in which one hemicord fails to neurulate and is exposed as a placode while the other, normally formed hemicord lies beneath, often obscured by normal appearing dura. A hemimyelomeningocele should be suspected when the placode lies slightly off midline and the patient has markedly asymmetrical sensorimotor function [8-10].

Some have advocated for resecting the neural placode at the time of initial closure for those patients without significant lower extremity function, and for those with significant kyphosis in whom a simultaneous restorative vertebrectomy may be undertaken [11]. Although one study noted improvements in bladder function after placode resection performed at the time of later scoliosis correction, other authors have pointed out that since the placode sometimes has underlying reflex (and occasionally voluntary) sensorimotor and/or bowel and bladder function, resecting the placode places these reflex functions at risk (for example converting a reflex bladder that can retain continence between catheterizations to a flaccid and continuously dribbling bladder) and note deterioration in approximately 8% [12,13,14]. We therefore do not generally resect the placode at the time of closure.

Once the placode is closed, attention is turned toward closing the arachnoid and dural layers. Again, since primary neurulation has not occurred at this level, the arachnoid and dura remain everted (think again of the lobster tail) and need to be elevated and brought together and closed in the midline. Some neurosurgeons advocate closing the arachnoid as a separate layer, whereas others simply close both layers together; there is no evidence that one technique is superior to the other. The arachnoid/dura is best identified laterally; an incision is made and the arachnoid/dura undermined and elevated from the underlying lumbodorsal fascia. Once the initial incision has been made and the proper plane identified, our technique is to place one blade of the scissors beneath, and the other atop the arachnoid/dura, oriented in a craniocaudal direction (Figure 4B). We then slide the scissors laterally until they stop at the edge of the arachnoid/dura, and then extend the incision circumferentially in this fashion. This technique ensures that the dural sac is as large as possible. Once the arachnoid/dura is dissected circumferentially, underlying bands of tissue can be sharply cut until one encounters yellow-brown fat in the epidural space. The arachnoid/dura can then be brought together and closed primarily in the midline (Figure 4C). Care should be taken to avoid strangulating the neural placode; if necessary, a patch graft may be used. The dura should be closed in a water tight fashion to minimize the risk of CSF leakage post-operatively.

The next stage of closure involves elevation and closure of the skin, with or without additional closure of the paraspinous musculature and fascia. Some have advocated elevating and closing the lumbodorsal musculature and fascia in an effort to both restore anatomical relationships (‘relocating’ the paraspinous muscles to their anatomical position posterior rather than anterior to the spinal cord), provide further protection against trauma, and further reduce the risk of post-operative CSF leak [15]. However, this may risk a greater blood loss and retroperitoneal injury. The technique involves a curved incision in the lumbodorsal fascia and the superficial portion of the underlying paraspinous muscles, undermining them laterally, drawing them together in the midline over the spinal canal. Removal of prominent lateral bony arches may sometimes facilitate closure of this layer. This may be more difficult than it looks, and care must be taken to avoid going too deep (and injuring the retroperitoneum). Once again, care should be taken not to strangulate the placode and dura.

The last task is to close the skin. The skin is undermined circumferentially around the defect, dissecting laterally to a greater degree than superiorly, and separating the skin from the underlying muscular fascia. This is best done with scissors using a combination of blunt and sharp dissection; one must be careful to identify the proper layer just superficial to the lumbodorsal fascia and posterior musculature, and avoid dissecting either deeper or more superficial which risks retroperitoneal or skin injury, respectively. The amount of skin elevation is variable, but needs to be sufficient to bring the skin edges together in the midline. This sometimes involves dissecting the skin to the posterior axillary line or even further (as far as the umbilicus) in difficult or large closures.

We always try to close the skin in a vertical fashion, which makes subsequent tethered cord surgery easier. The skin virtually always comes together with some degree of tension; often this would make a plastic surgeon nervous, but we have found that this tension rarely results in ischemia. The dysplastic skin attached to the flaps should be left until the final stages of the closure. The skin flaps are brought together, and the Scarpa’s fascia and deep dermis are identified along the underside of the normal skin, lateral to the thinner dysplastic skin that will subsequently be trimmed. The Scarpa’s and deep dermis are closed in a single layer. Once closed, the overlying dysplastic skin can be trimmed in the midline, and the remaining skin edges closed either with subcuticular or full thickness skin sutures. The skin under tension may appear blanched initially, but usually regains its capillary refill within several minutes. We have used a small amount of topical nitroglycerin paste on this blanched skin to allow vasodilation; although there is no published research that examines the efficacy of this practice, the skin does seem to ‘pink up’ afterward, and we have had no complications from this [16].

Complications of primary skin closure may include skin breakdown or ischemia, and CSF leak. Skin breakdown is usually presaged by ischemic looking or black areas in the surrounding tissues. This is usually followed by skin necrosis with eschar formation. The eschar can often be left in place, as it provides a barrier while the underlying tissues granulate secondarily. Once the eschar falls off, the underlying granulation may allow healing by secondary intent, thereby avoiding subsequent operations. CSF leak can be treated as usual with additional local sutures; however, repeated CSF leak should signal the development of hydrocephalus and may require a shunt.

 

Plastic surgical adjuncts to myelomeningocele closure

Although most MMC can be closed primarily, as many as 25%, particularly larger defects > 5 cm in mediolateral width, may benefit from adjunctive plastic surgical techniques. A number of techniques have been described, depending on the size and location of the defect along the craniocaudal axis [17]. It is not our purpose here to advocate for one or another technique, but to discuss each.

There are a number of described cutaneous, musculocutaneous, and fasciocutaneous flaps that may provide tension free closure and soft tissue padding while maintaining the vascular supply to prevent flap ischemia and eventual breakdown. The choice of flap depends largely upon the geometry and size of the defect. Advancement and rotational cutaneous flaps are generally selected based on defect shape as well as the availability of local donor tissue; options include quadruple perforator buttery flaps, keystone design perforator island flaps, Limberg flaps, Emsen flaps, O-S flaps, binary VY flaps and others. Musculocutaneous flaps may use latissimus dorsi, gluteus, and trapezius pedicles based on their vascular supplies. Additional adjuncts may include skin grafting or acellular dermal matrix.

**Figure 5:**
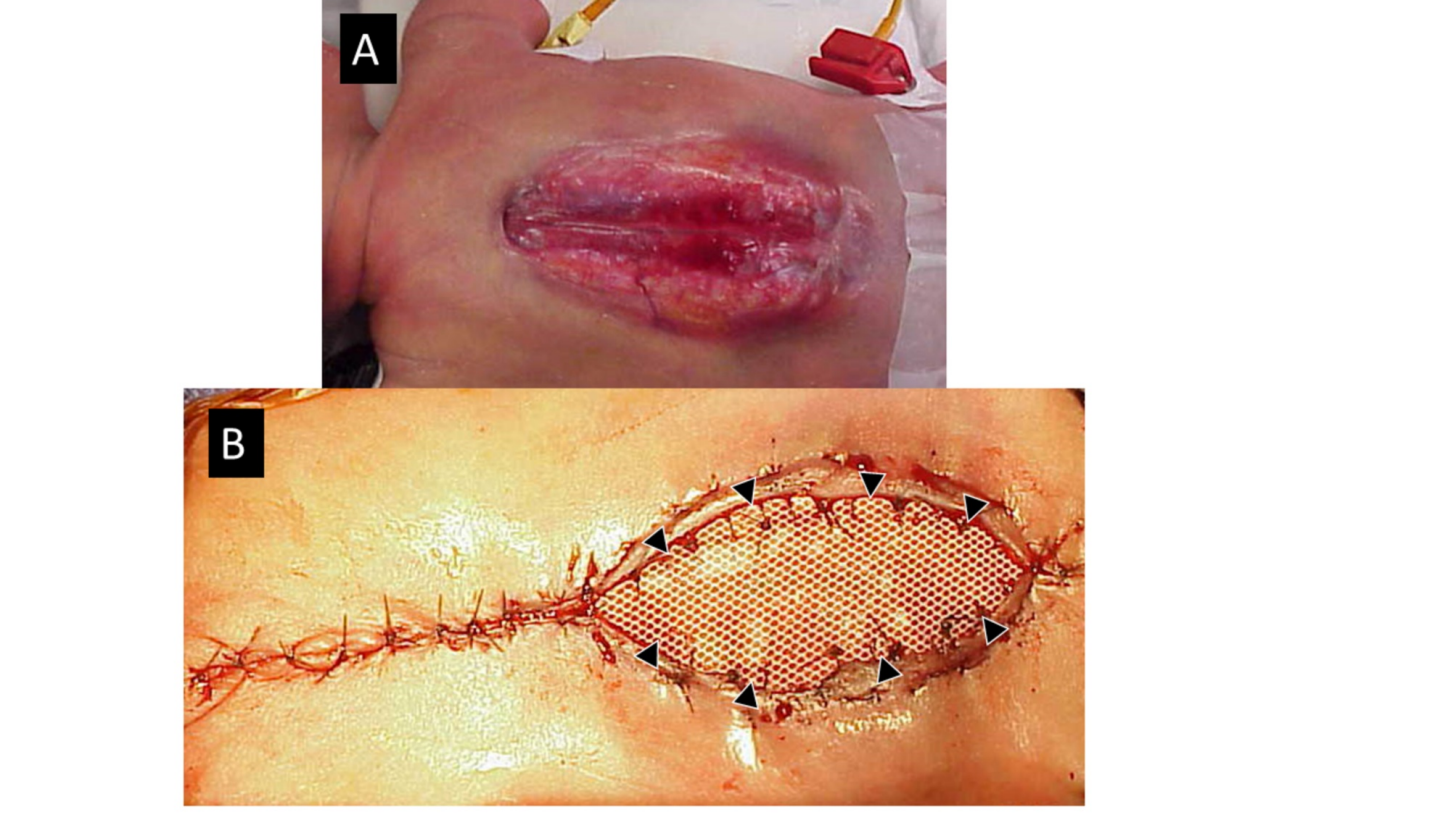
A huge MMC (T4 to sacrum) closed with silastic mesh A. The defect covers virtually the entire back. B. The upper end has been closed primarily and a silastic mesh has been sewn to the undersurface of the skin edges at the caudal end (arrowheads). This mesh is subsequently plicated every 2-3 days on the ward to progressively draw the skin edges together.

For very large flaps, another technique that we have used involves a silastic sheet that is cut to a size that allows it to be sutured to the undersurface of the skin lateral, superior, and inferior to the skin edges without significant tension (Figure 5). We suture the sheet to Scarpa’s fascia and the deep dermis of the more lateral skin, thereby covering the skin defect. The center of the silastic sheet is then progressively plicated with sutures placed along a vertical line every 2-4 days on the ward, drawing the skin edges progressively together over time. Once the lateral skin edges are overlapping, the infant can be returned to the operating room, the silastic sheet removed, and the skin edges closed primarily.

## Discussion

A large number of previous publications have focused on various aspects of care for children with MMC, including technical nuances on the initial closure. Although these are helpful for the neurosurgeon, both pre- and intra-operative findings may be encountered that are difficult to understand completely without a working knowledge of the embryology of early neural tube closure. This understanding allows the neurosurgeon to better interpret both the anatomy of the placode and properly identify its variants.

An embryological approach allows the surgeon to better understand the neural placode as a defect in neuro tube closure with persistent attachment of the neuroectoderm to the surrounding cutaneous ectoderm (skin), and that the MMC is by definition always an open malformation. This also allows a better understanding of the relationships between the placode and nerve roots, the surrounding arachnoid and dura, the posterior bone elements, the muscle and fascia, and the skin as layers of an onion that have been everted from their normal embryological positions. Knowing the independence of primary and secondary neurulation allows the surgeon to recognize the possibility that there may be a tethering filum terminale that should be addressed. Finally, understanding the circumstances and features of hemimyelomeningocele and split cord malformations allows these unusual malformations to be recognized and addressed at the time of primary closure.

## Conclusions

Pediatric neurosurgeons and all who undertake MMC repair should keep basic embryological principles in mind when approaching MMC repair. Knowing these principles creates a better understanding of the anatomy of the placode and its relationship with the surrounding structures; the surgeon armed with this information may be better prepared to handle routine MMC closure and identify and properly address unusual circumstances that may be encountered.
